# Hyponatremia caused by water intoxication: successful treatment of psychiatric disturbances with olanzapine and fluoxetine

**DOI:** 10.1093/omcr/omaa127

**Published:** 2021-01-23

**Authors:** Maris Taube

**Affiliations:** 1 Department of Psychiatry and Narcology, Riga Stradins University, Riga, LV 1005, Latvia; 2 Riga Center of Psychiatry and Narcology, Depression and Crisis Unit Riga, LV 1064, Latvia

## Abstract

Hyponatremia is a frequent, yet often unrecognized result of water intoxication caused by psychogenic polydipsia in patients with psychiatric disorders such as schizophrenia and anorexia nervosa. The consequences of hyponatremia may include cerebral edema with tonic–clonic seizures and, in extreme cases, death. In cases of hyponatremia seen in psychiatric practices, the use of psychotropic drugs is often necessary to address both the underlying psychiatric problem and reduce the hyponatremia. Therefore, a patient’s clinical condition, the risk of side effects, the possible effect of the medication on hyponatremia and a history of prior medication use should be considered when selecting appropriate psychotropics. The present clinical case details the beneficial effects of olanzapine and fluoxetine in treating a patient with anorexia nervosa and body dysmorphic disorder experiencing acute hyponatremia, and the stable effect the medications achieved over a period of 2.5 years of maintenance therapy.

## INTRODUCTION

Water intoxication is an uncommon condition that is typically caused by primary or psychogenic polydipsia. It occurs in 15–25% of patients with chronic mental diseases [1]. Psychogenic polydipsia, beside other reasons (‘iatrogenic’, sarcoidosis, brain injury), may cause by psychotropic medications, e.g. all antidepressants, antipsychotics (olanzapine, aripiprazole, clozapine and quetiapine), mood stabilizers (carbamazepine/oxcarbamazepine, valproate and lamotrigine) [2, 3]. Several risk factors predispose hyponatremia in patients with polydipsia: chronicity of polydipsia, psychosis, acute stress reactions, smoking, drugs and acute infections [3]. This case report details the occurrence of water intoxication in a psychiatric patient to increase clinical awareness regarding psychogenic polydipsia as a causal factor in hyponatremia.

**Table 1:** Case treatment history (hospitalisation’s descriptions, treatment, treatment results, clinical investigations and blood tests results)

												Blood test 19/05/2020	Units	Reference range
No of hospitalisations	1	2	3	4	5	6	7	8	9	10	11			
Treatment place	Psychiatric hospital	General hospital	General hospital	Psychiatric hospital	Psychiatric hospital	General hospital	General hospital	General hospital	General hospital	General hospital	General hospital			
Treatment period	24/08/2005–29/01/2006	5/03/2014–11/03/2014	19/01/2015–18/03/2015	18/03/2015–17/04/2015	29/02/2016–05/04/2016	17/05/2016–25/05/2016	17/02/2017–03/03/2017	13/03/2017–17/03/2017	16/10/2017–19/10/2017	15/01/2018–26/01/2018	12/03/2018–20/04/2018			
Main diagnosis/reason for hospitalization	Anorexia nervosa (F50.00 ICD 10)	Malnutrition, amenorrhea, psychogenic polydipsia	Anorexia nervosa (F50.00 ICD 10), malnutrition, osteoporosis, fracture os pubis	Delusional dysmorphophobia (F22.0 ICD 10)	Delusional dysmorphophobia (F22.0 ICD 10)	Psychogenic polydipsia, seizure (epileptic type)	Psychogenic polydipsia (25 liter liquid per day)	Psychogenic polydipsia, seizure (epileptic type)	Symptomatic hypoglycaemic episodes, psychogenic polydipsia, symptomatic hyponatremia	Psychogenic polydipsia (16–17 liter liquid per day), symptomatic cardiomyopathy	Haemophilus parainfluenza lung infection, Psychogenic polydipsia (16 liter liquid per day), symptomatic cardiomyopathy, urostasis			
BMI	9.2	13.4	12	16	13.8		17.3					24		
White cell count		5.48	5.84			7.75	4.06	7.66	4.97	4.31	7.15	7.64	×10^9^/L	4.0–11.0
Platelet		296	277			247	232	203	291	270	343	327	×10^9^/L	150–400
Sodium		130 (139 after 5 h liquid intake restriction)	132		122	120.96	117	128	120	112	108.25	131	mmol/l	135–145
Potassium		4.65	3.71			3.51	4.46	3.55	4.92	4.15	4.2	5.42	mmol/l	3.5–5.0
Calcium		2.38				2.41	2.19	2.21	2.36	2.44		2.55	mmol/l	2.12–2.63
ALAT		20	21			12		16.86	21	21	26	25	U/L	<45
GGT		37								30			U/L	<55
Creatinine		43	28			31.81	25	23.69	31	31	24	42	μmol/L	45–90
Total protein		68.5	46.14						62.4		58		g/L	60–80
Glucose		3.95	5.03			6.56		6.03	4.29	5.79	4.8	4.63	mmol/L	3.3–5.89
EKG		Norm	Sinus tachycardia			Sinus tachycardia, nonspecific ST–T changes	Norm			QT prolongation				
CT cranium						No acute pathologies	No acute pathologies			No acute pathologies				
MRI cranium			Atrophies											
EEG						Subcortical encephalopathy, epileptic activity								
Main treatment recommendations	Haloperidol 2 mg per day, clomipramine 62.5 mg per day, paroxetine 20 mg per day	Diet, reduction of fluid intake, acidum valproicum/Na valproas 300 mg per day	Physiotherapy, nutritional supplements, treatment in psychiatric hospital	Psychotherapy, art therapies, physiotherapy ergotherapy, nutritional supplements, fluoxetine 40 mg per day, quetiapine 125 mg per day, gabapentin 600 mg per day, lorazepam 1,25 mg per day	Psychotherapy, art therapies, physiotherapy ergotherapy, nutritional supplements, fluoxetine 40 mg per day, quetiapine 125 mg per day, olanzapine 10 mg per day,	Diet, reduction of fluid intake, acidum valproicum/Na valproas 300 mg per day, fluoxetine 20 mg per day, quetiapine 150 mg per day, clomipramine 20 mg per day, olanzapine 10 mg per day	Diet, reduction of fluid intake, quetiapine 75 mg per day, fluoxetine 20 mg per day, clomipramine 20 mg per day	Diet, reduction of fluid intake, quetiapine 75 mg per day, fluoxetine 20 mg per day, risperidone 2 mg per day, acidum valproicum/Na valproas 300 mg per day	Diet, reduction of fluid intake, fluoxetine 20 mg per day, olanzapine 10 mg per day	Diet, reduction of fluid intake, fluoxetine 20 mg per day, olanzapine 10 mg per day, nebivolol 2.5 mg per day, Ca citratum, Ramipril 5 mg per day	Diet, reduction of fluid intake, fluoxetine 20 mg per day, olanzapine 10 mg per day, nebivolol 2.5 mg per day, Ca citratum, Ramipril 5 mg per day			
Result/continuity of treatment	BMI 16.8, improvement, episodic treatment with quetiapine, aripiprazole, risperidone, quit medicines (no medical reports available)	Improvement, episodic, unregular medicine use (zuclopenthixol 20 mg, per day, haloperidol 4 mg per day)	BMI 16, the patient is able to walk	Improvement, low inside, quit medicines	Improvement, low inside	Improvement, low inside, excessive use of liquids. Endocrinologists hypothesis about causal role of antipsychotics in polydipsia and hyponatraemia	Improvement, low inside, excessive use of liquids	Improvement, low inside, excessive use of liquids	Improvement, low inside, quit medicines, excessive use of liquids	Improvement, low inside, excessive use of liquids, the patient didn’t quit medicines, mentally improved, live independently, work	Improvement, regular visits to psychiatrist, regular usage of medicines, mentally improved, live independently, work, no future hospitalisations caused by hyponatremia			

## CASE REPORT

The patient is a 34-year-old female whose health problems began at the age of 14 when she perceived herself as overweight. By the age of 17 improvements had been achieved through psychotherapy and pharmacotherapy. During this period, the patient was excessively consuming fluid to reduce her vomiting episodes and suppress appetite. Between 2014 and 2018, she was hospitalized 10 times for malnutrition, amenorrhea, osteoporosis, secondary macrocytic anemia, as well as pronounced thirst, excessive fluid intake and plasma sodium level < 130 mmol/L (Table 1). However, she continued to have non-adjusting thoughts of being overweight.

Two times (2015 and 2016) her treatment and rehabilitation continued in a psychiatric clinic where she received treatment comprising psychotherapy, art therapy, physiotherapy, ergotherapy and pharmacotherapy. In 2016 (hospitalization no 5) combination of fluoxetine at 40 mg/day, and olanzapine at 10 mg/day were recommended by psychiatrist. Improvement was achieved; but the patient could not cooperate with her psychiatrist successfully, denied psychiatric cause of hyponatremia and did not use psychotropic medicines regularly.

In 2016 (hospitalization no 6), the patient was hospitalized for seizures, confusion and disorientation. An examination revealed that she had been consuming more than 6 L (till 25) of water per day. Her endocrinologist was inclined to consider that the polydipsia was likely caused by her prescribed antipsychotic medications. In 2017, the patient was hospitalized three more times (hospitalization no 7, 8 and 9) for seizures because of hypoglycemia. In beginning of 2018 (after hospitalizations no 10), the patient was convinced by psychiatrist to return to a regular every day medication regime—combination of fluoxetine at 20 mg/day, and olanzapine at 10 mg/day. After regular use of mentioned combination of psychotropic medications (at least 4–6 months while medicines started to work), stopped her thoughts of being overweight, the patient gained weight and is currently healthy. Although her plasma sodium levels continue to remain slightly reduced at 130–134 mmol/L, her condition has been stable for 2.5 years.

## DISCUSSION

Water intoxication is an uncommon condition that is frequently caused by psychogenic polydipsia [4]. In this case, our patient suffered from anorexia nervosa (F50.00), which had developed into delusional body dysmorphic disorder (F22.0) by the time of her water intoxication onset. Seizures, which were observed in our patient, are reliably associated with cerebral edema [5] and are characteristic in cases of severe hyponatremia. Some, often unnoticed by physicians, symptoms like falls, gait instability and attention impairments are first signs of hyponatremia [6–7].

Treatment of our patient’s hyponatremia was complicated by the skepticism of some of her endocrinologist with regard to the potential psychogenic etiology of her hyponatremia.

Hyponatremia may be caused by selective serotonin reuptake inhibitors, and fluoxetine is considered to be an especially high-risk medication for the condition [8]; however, it was administered in this instance as the patient had previously used it with good results.

Cases of hyponatremia have also been associated with the use of antipsychotics [2, 9]. Conversely, it has also been reported that atypical antipsychotics can help in cases when hyponatremia is associated with psychogenic polydipsia [10]. In these instances, risperidone and olanzapine may demonstrate superior effects [8]. In the current case, olanzapine effectively reduced the patient’s delusional thoughts regarding her appearance. This was possibly related to the primary effectiveness of olanzapine on psychotic disorders and, specifically, on psychogenic polydipsia.

For our patient, olanzapine and fluoxetine were found to be effective treatments for psychogenic polydipsia.

## CONFLICT OF INTEREST STATEMENT

None declared.

## FUNDING

No funding was received for this study.

## ETHICAL APPROVAL

This research meets ethical guidelines and adheres to the local legal requirements.

## CONSENT

Written informed consent was obtained from the patient for publication of this case report.

## GUARANTOR

Maris Taube.

## Supplementary Material

Table1_Table1-_Case_treatment_history_omaa127
