# Supplementary material for: Hyponatremia caused by water intoxication: successful treatment of psychiatric disturbances with olanzapine and fluoxetine
Source: Oxf Med Case Reports. 2021 Jan 23;2021(1):omaa127. doi: 10.1093/omcr/omaa127 (PMC7846081; doi:10.1093/omcr/omaa127)
Supplement: Table1_Table1-_Case_treatment_history_omaa127 [file table1_table1-_case_treatment_history_omaa127.docx]

Table1: Case treatment history (hospitalisation’s descriptions, treatment, treatment results, clinical investigations and blood tests results)

|  |  |  |  |  |  |  |  |  |  |  |  | Blood test 19/05/2020 | Units | Reference range |
| --- | --- | --- | --- | --- | --- | --- | --- | --- | --- | --- | --- | --- | --- | --- |
| No of hospitalisations | 1 | 2 | 3 | 4 | 5 | 6 | 7 | 8 | 9 | 10 | 11 |  |  |  |
| Treatment place | Psychiatric hospital | General hospital | General hospital | Psychiatric hospital | Psychiatric hospital | General hospital | General hospital | General hospital | General hospital | General hospital | General hospital |  |  |  |
| Treatment period | 24/08/2005-29/01/2006 | 5/03/2014-11/03/2014 | 19/01/2015-18/03/2015 | 18/03/2015-17/04/2015 | 29/02/2016-05/04/2016 | 17/05/2016-25/05/2016 | 17/02/2017-03/03/2017 | 13/03/2017-17/03/2017 | 16/10/2017-19/10/2017 | 15/01/2018-26/01/2018 | 12/03/2018-20/04/2018 |  |  |  |
| Main diagnosis/  reason for hospitalisation | Anorexia nervosa (F50.00 ICD 10) | Malnutrition, amenorrhea, psychogenic polydipsia | Anorexia nervosa (F50.00 ICD 10), malnutrition, osteoporosis, fracture os pubis | Delusional dysmorphophobia (F22.0 ICD 10) | Delusional dysmorphophobia (F22.0 ICD 10) | Psychogenic polydipsia, seizure (epileptic type) | Psychogenic polydipsia (25 litre liquid per day) | Psychogenic polydipsia, seizure (epileptic type) | Symptomatic hypoglycaemic episodes, psychogenic polydipsia, symptomatic hyponatremia | Psychogenic polydipsia (16-17 litre liquid per day), symptomatic cardiomyopathy | Haemophilus parainfluenza lung infection, Psychogenic polydipsia (16 litre liquid per day), symptomatic cardiomyopathy, urostasis |  |  |  |
| BMI | 9.2 | 13.4 | 12 | 16 | 13.8 |  | 17.3 |  |  |  |  | 24 |  |  |
| White cell count |  | 5.48 | 5.84 |  |  | 7.75 | 4.06 | 7.66 | 4.97 | 4.31 | 7.15 | 7.64 | x10^9^/L | 4.0–11.0 |
| Platelet |  | 296 | 277 |  |  | 247 | 232 | 203 | 291 | 270 | 343 | 327 | x10^9^/L | 150–400 |
| Sodium |  | 130 (139 after 5h liquid intake restriction) | 132 |  | 122 | 120.96 | 117 | 128 | 120 | 112 | 108.25 | 131 | mmol/l | 135–145 |
| Potassium |  | 4.65 | 3.71 |  |  | 3.51 | 4.46 | 3.55 | 4.92 | 4.15 | 4.2 | 5.42 | mmol/l | 3.5–5.0 |
| Calcium |  | 2.38 |  |  |  | 2.41 | 2.19 | 2.21 | 2.36 | 2.44 |  | 2.55 | mmol/l | 2.12–2.63 |
| ALAT |  | 20 | 21 |  |  | 12 |  | 16.86 | 21 | 21 | 26 | 25 | U/L | <45 |
| GGT |  | 37 |  |  |  |  |  |  |  | 30 |  |  | U/L | <55 |
| Creatinine |  | 43 | 28 |  |  | 31.81 | 25 | 23.69 | 31 | 31 | 24 | 42 | μmol/l | 45–90 |
| Total protein |  | 68.5 | 46.14 |  |  |  |  |  | 62.4 |  | 58 |  | g/l | 60–80 |
| Glucose |  | 3.95 | 5.03 |  |  | 6.56 |  | 6.03 | 4.29 | 5.79 | 4.8 | 4.63 | mmol/l | 3.3–5.89 |
| EKG |  | Norm | Sinus tachycardia |  |  | Sinus tachycardia, nonspecific ST–T changes | Norm |  |  | QT prolongation |  |  |  |  |
| CT cranium |  |  |  |  |  | No acute pathologies | No acute pathologies |  |  | No acute pathologies |  |  |  |  |
| MRI cranium |  |  | Atrophies |  |  |  |  |  |  |  |  |  |  |  |
| EEG |  |  |  |  |  | Subcortical encephalopathy, epileptic activity |  |  |  |  |  |  |  |  |
| Main treatment recommendations | Haloperidol 2 mg per day, clomipramine 62.5 mg per day, paroxetine 20 mg per day | Diet, reduction of fluid intake, acidum valproicum/Na valproas 300 mg per day | Physiotherapy, nutritional supplements, treatment in psychiatric hospital | Psychotherapy, art therapies, physiotherapy ergotherapy, nutritional supplements, **fluoxetine 40 mg per day,** quetiapine 125 mg per day, gabapentin 600 mg per day, lorazepam 1,25 mg per day | Psychotherapy, art therapies, physiotherapy ergotherapy, nutritional supplements, **fluoxetine 40 mg per day**, quetiapine 125 mg per day, **olanzapine 10 mg per day**, | Diet, reduction of fluid intake, acidum valproicum/Na valproas 300 mg per day, **fluoxetine 20 mg per da**y, quetiapine 150 mg per day, clomipramine 20 mg per day, **olanzapine 10 mg per day** | Diet, reduction of fluid intake, quetiapine 75 mg per day, **fluoxetine 20 mg per day,** clomipramine 20 mg per day | Diet, reduction of fluid intake, quetiapine 75 mg per day, **fluoxetine 20 mg per day**, risperidone 2 mg per day, acidum valproicum/Na valproas 300 mg per day | Diet, reduction of fluid intake, **fluoxetine 20 mg per day**, **olanzapine 10 mg per day** | Diet, reduction of fluid intake, **fluoxetine 20 mg per day**, **olanzapine 10 mg per day,** nebivolol 2.5 mg per day, Ca citratum, Ramipril 5 mg per day | Diet, reduction of fluid intake, **fluoxetine 20 mg per day**, **olanzapine 10 mg per day,** nebivolol 2.5 mg per day, Ca citratum, Ramipril 5 mg per day |  |  |  |
| Result/ continuity of treatment | BMI 16.8, improvement, episodic treatment with quetiapine, aripiprazole, risperidone, quit medicines (no medical reports available) | Improvement, episodic, unregular medicine use (zuclopenthixol 20 mg, per day, haloperidol 4 mg per day) | BMI 16, the patient is able to walk | Improvement, low inside, quit medicines | Improvement, low inside | Improvement, low inside, excessive use of liquids. Endocrinologists hypothesis about causal role of antipsychotics in polydipsia and hyponatraemia | Improvement, low inside, excessive use of liquids | Improvement, low inside, excessive use of liquids | Improvement, low inside, quit medicines, excessive use of liquids | Improvement, low inside, excessive use of liquids, the patient didn’t quit medicines, mentally improved, live independently, work | Improvement, regular visits to psychiatrist, regular usage of medicines, mentally improved, live independently, work, no future hospitalisations caused by hyponatremia |  |  |  |
